# Preliminary studies on isolates of *Clostridium difficile* from dogs and exotic pets

**DOI:** 10.1186/s12917-018-1402-7

**Published:** 2018-03-09

**Authors:** Sara Andrés-Lasheras, Inma Martín-Burriel, Raúl Carlos Mainar-Jaime, Mariano Morales, Ed Kuijper, José L. Blanco, Manuel Chirino-Trejo, Rosa Bolea

**Affiliations:** 1 Departamento de Patología Animal, Facultad de Veterinaria, Instituto Agroalimentario de Aragón - IA2 - (Universidad de Zaragoza-CITA), 50013 Zaragoza, Spain; 2 Laboratorio de Genética Bioquímica (LAGENBIO), Facultad de Veterinaria, Instituto Agroalimentario de Aragón - IA2 - (Universidad de Zaragoza-CITA), Zaragoza, Spain; 3 Laboratorios Albéitar, Zaragoza, Spain; 4 Department of Medical Microbiology, Centre of Infectious Diseases, Leiden University Medical Centre, Leiden, The Netherlands; 5 Departamento de Sanidad Animal, Facultad de Veterinaria, Universidad Complutense, Madrid, Spain; 6 Department of Veterinary Microbiology, Western College of Veterinary Medicine, University of Saskatchewan, Saskatoon, Canada

**Keywords:** *Clostridium difficile*, Dog, Exotic, Metronidazole-resistant, PCR-ribotyping, MLST

## Abstract

**Background:**

*Clostridium difficile* infection (CDI) is recognised as an emerging disease in both humans and some animal species. During the past few years, insights into human CDI epidemiology changed and *C. difficile* is also considered as an emerging community-acquired pathogen. Certain ribotypes (RT) are possibly associated with zoonotic transmission. The objective of this study was to assess the presence of *C. difficile* in a population of pets and to characterise the isolates.

**Results:**

Faecal samples from a total of 90 diarrhoeic dogs and 24 from exotic animal species (both diarrhoeic and non-diarrhoeic) were analysed. *Clostridium difficile* was isolated from 6 (6.7%) dogs and one reptile sample (4.2%). Four (66.7%) of the six dog strains were capable of producing toxins. Four known different RTs were detected in dogs (010, 014, 123 and 358) and a new one was found in a faecal sample of an exotic animal. This new RT isolate was negative for all toxin genes tested and belonged to sequence type 347 which has been proposed as a Clade-III member. Importantly, two dog strains showed a stable resistance to metronidazole (initial MIC values: 128 and 48 μg/ml).

**Conclusions:**

The results obtained in this study suggest the implementation of antimicrobial susceptibility surveillance programs to assess the prevalence of metronidazole resistance in dogs; molecular studies to elucidate *C. difficile* metronidazole resistance mechanisms are warranted. Based on the similarity between the ribotypes observed in dogs and those described in humans, the zoonotic transmission should be further explored. Furthermore, exotic animals have shown to harbor uncommon *C. difficile* strains which require further genomic studies.

**Electronic supplementary material:**

The online version of this article (10.1186/s12917-018-1402-7) contains supplementary material, which is available to authorized users.

## Background

*Clostridium difficile* is a bacterium capable of producing enteric disease in different animal species included humans. Toxigenic *C. difficile* strains are the most common cause of antibiotic-associated diarrhoea in people from developed countries through the synthesis of toxins A and B, its main virulence factors [1]. The epidemiology of *C. difficile* has changed in the last 15 years and it is now recognised as an emerging pathogen in both humans and animals. It is also considered an emerging community-acquired pathogen likely associated with a zoonotic and/or foodborne transmission [2].

Animals are an important source of many infectious diseases for humans. About 75% of emerging infectious diseases are zoonoses [3], and it is thought that pets could be implicated in the transmission of *C. difficile* to humans since similar genotypes have been recovered from them and humans [4]. Among the strains isolated from dogs, there are several ribotypes of international interest, such as RT078, RT014/020 and RT045 [5]. Likewise, exotic animals can act as vectors of many zoonotic diseases, including enteric diseases, and several zoonotic outbreaks have been associated with the trade of this type of animal species (either in a legal or illegal way) as their international trade has increased recently [6]. However, there is limited information about toxigenic *C. difficile* carriage in exotic animals in the scientific literature [7].

Dogs can develop diarrhoea due to different bacteria or virus infections (e.g. *Salmonella* spp., *Campylobacter* spp., *Clostridium perfringens*, *C. difficile*, or coronavirus), intestinal parasites (e.g. *Giardia* spp.), nutritional factors, inflammation, allergies or neoplasia [8, 9]. So far, the role of *C. difficile* in canine enteric disease is still unclear due to the presence of toxigenic strains or their toxins in asymptomatic animals and the failure to reproduce CDI in healthy dogs with and without antibiotic treatment [9, 10]. It can be isolated from 0 to 57% of healthy dogs with no diarrhoea [11]. However, there are several studies which have reported an association between the presence of *C. difficile* toxins in faeces with diarrhoea, as well as with outbreaks of haemorrhagic diarrhoea in veterinary hospitals [9, 12–14]. But it is still unknown whether *C. difficile* represents an opportunistic pathogen, or simply a fortuitous finding in this animal species [10].

There is limited information about antimicrobial susceptibilities of *C. difficile* strains obtained from dogs or exotic animal species [7, 15]. Metronidazole, and less frequently vancomycin, are used to treat CDI in dogs following the recommendations for human episodes [16]. These drugs are also commonly used to treat different kinds of exotic animal infections as well. The isolation of metronidazole-resistant strains or strains showing low susceptibility to this drug is growing in both humans and animals [17], particularly in ribotypes 010 and 001 [18, 19]. In addition, resistance to metronidazole is heterogeneous and therefore *C. difficile* can show reduced susceptibility to this drug, which can be related to recurrent CDI cases [20].

The objective of this study was to assess the presence of *C. difficile* in a population of diarrhoeic dogs and exotic animal species (both diarrhoeic and non-diarrhoeic), and to characterise the *C. difficile* isolates by the presence of toxin genes, their PCR-ribotype and toxinotype, and also their antimicrobial susceptibility pattern.

## Methods

### Sampling

Diarrhoeic stool samples from dogs, submitted to a veterinary diagnostic laboratory located in the Zaragoza, NE of Spain were used. The samples were collected between October 2011 and November 2012. All of them were analysed for the presence of *C. difficile* by microbiological culture in our laboratory. The specimens were collected using commercial swabs with Amies transport medium (Deltalab, Barcelona, Spain) by veterinary staff at the time of the animal clinical examination. The samples originated from primary veterinary clinics located at different Spanish regions (Table 1). All of them were sent under refrigerated conditions to the diagnostic laboratory, arrived within the 24 h after collection, and were kept at − 80 °C until *C. difficile* culture was performed. In addition to *C. difficile*, faecal samples were analysed for other enteric pathogens upon request of the veterinarian in charge of the case (analysis carried out in the diagnostic laboratory; data not published). Dogs were classified by their age (0–4 months puppy; 5–12 months juvenile; 13–72 months adult; ≥73 months mature), gender, breed, location and presence/absence of dysbiosis. In addition, other laboratory data were recorded for each case when available.

**Table 1:** Characteristics of the dog population included in the study

Variable	Number samples (%)	Number positive samples (%) to CD	*p* ^a^	Number toxigenic CD strains (%)^b^
Gender			1	
Female	47 (52.2)	3 (6.4)		2 (4.2)
Male	43 (47.8)	3 (7)		2 (4.6)
Age^c^			0.42	
0–4 m	25 (27.8)	2 (8)		2 (8)
5–12 m	21 (23.3)	0		0
13–72 m	21 (23.3)	2 (9.5)		2 (9.5)
≥ 73 m	9 (10)	0		0
Spanish geographical region			0.77	
North	36 (40)	2 (5.6)		2 (5.6)
Northeast	42 (46.7)	3 (7.1)		2 (4.8)
Centre	7 (7.8)	1 (14.3)		0
Southeast	5 (5.5)	0		0
Season			0.71	
Winter	31 (34.4)	1 (3.2)		1 (3.2)
Spring	9 (10)	1 (11.1)		1 (11.1)
Summer	3 (3.3)	0		0
Fall	47 (52.2)	4 (8.5)		2 (4.3)
Breed^d^			0.63	
Large	40 (44.4)	2 (5)		1 (2.5)
Medium	29 (32.2)	2 (6.9)		2 (6.9)
Small	13 (14.4)	0		0
Dysbiosis			0.66	
No	28 (31.1)	1 (3.6)		0
Yes	62 (68.9)	5 (8.1)		4 (6.5)

*CD Clostridium difficile*; ^a^Univariable logistic regression; ^b^percentages calculated from the total number of samples; ^c^age unknown for 14 samples (two of them are positive to non-toxigenic *C. difficile*); ^d^breed unknown for eight samples (one of them is positive to toxigenic *C. difficile* and another one to non-toxigenic *C. difficile*)

Additionally, faecal samples were cultured from exotic animals from a veterinary clinic laboratory specialised in that kind of animals located in Barcelona, NE of Spain. The sampling period covered June and July 2013. The specimens derived from diarrhoeic and non-diarrhoeic animals. Each sample was taken by the veterinary staff during the examination of the animals and submitted to our laboratory under refrigerated conditions within the first 24 h after collection. As in the dog’s study, the samples were kept at − 80 °C until the culture for *C. difficile* was performed. Exotic animals were identified by species and grouped into three main animal categories (i.e. reptiles, small mammals and birds). No other data were available except being sick or not.

### Bacterial culture and strain characterisation

The isolation of *C. difficile*, molecular characterisation of the strains obtained (i.e. *tpi* housekeeping and toxin genes detection by PCR, identification of non-toxigenic strains, and PCR-ribotyping) and antimicrobial susceptibility testing was performed as described elsewhere [21]. The variability of the genes coding for toxins A and B (A3 and B1 fragments respectively) of toxigenic strains obtained was assessed through toxinotyping by PCR-RFLP [22]. Besides, those strains which showed unexpected results, i.e. negative PCR results for *tcdA* and *tcdB* genes and also non-toxigenic assay, were further studied by toxinotyping as well (A1, A2, A3, B1, B2 and B3 fragments) to test the possible presence of toxin gene fragments.

Multilocus sequence typing (MLST) technique was used for characterisation of strains which did not belong to a known PCR ribotype. By this technique, seven housekeeping loci are studied by PCR and subsequent sequenced, providing a sequence type (ST) profile and a clade [23] based on these genes alleles [24]. A maximum likelihood tree with 1000 bootstrap replicates was constructed based on the Kimura 2-parameter model [25]. A total of 37 strains were used for this purpose: one from this study (E6), 17 from previous works carried out by our team (Hu, RC and RF isolates; data not published for Hu isolates) [26], and 19 from the PubMLST database to provide a context for *C. difficile* population (ST1, ST3, ST5, ST11, ST32, ST37–39, ST41, ST67, ST96, ST122, ST177–181, ST200 and ST206). Initial tree(s) for the heuristic search were obtained by applying the Neighbour-Joining method to a matrix of pairwise distances estimated using the Maximum Composite Likelihood approach. Evolutionary analyses were conducted in MEGA7 [27].

Additionally, seven serial passages over 14 days were performed on *Brucella* blood agar plates without antibiotics in order to assess the stability of the initial metronidazole-resistant isolates, i.e. those which showed a breakpoint ≥32 μg/ml [28]. Then, the MIC to metronidazole was tested again by Etest as described above. Resistance was considered stable when the MIC of metronidazole against *C. difficile* remained (within ±1 dilution) after the passages. Resistance was considered unstable when resistant strains became susceptible (˂32 μg/ml) after the passages.

### Statistical analysis

The prevalence of *C. difficile* was estimated for dogs and exotic pets separately. When possible, comparisons of *C. difficile* prevalence among the factors considered for dogs were assessed by univariable logistic regression. Thus, the outcome variable was the presence/absence of *C. difficile* and the explanatory variable was the corresponding factor (age, gender, breed, location and dysbiosis). Significance was set at *p*-values ≤0.05.

## Results

### Bacterial isolation and molecular characterization

Faecal samples from a total of 90 dogs were analysed. The samples came from 42 different veterinary clinics located in 11 different Autonomous Communities in Spain, most of them (86.7%) located in the north/northeast of the country. The median age of dogs was 9.5 months (range 1–156) and 52.2% of the animals were females. A total of 29 dog breeds were included and grouped as large (e.g. German shepherd), medium (e.g. Beagle) and small size (e.g. Yorkshire). *Clostridium difficile* was isolated from 6 (6.7%) out of the 90 dogs analysed. All these results are summarised in Table 1. Four (66.7%) strains with toxin genes (Table 2) yielded an A + B + CDT- genotype in all cases. Non-toxigenic strains showed a positive result with lok3/1 primers. Four different ribotypes were detected, and all toxigenic strains belonged to toxinotype 0 (Table 2).

**Table 2:** Molecular characterization of *Clostridium difficile* isolates

ID^a^	Toxin genes	NTS^b^	Toxinotype	RT	ST	MIC_0_/MIC_S_ to MZ^c^
D18	*tcdA*, *tcdB*	NA	0	014	–	–
D21	–	+	NA	123	–	–
D24^d^	*tcdA*, *tcdB*	NA	0	014	–	128/96
D57	*tcdA*, *tcdB*	NA	0	358	–	–
D66	*tcdA*, *tcdB*	NA	0	014	–	–
D83^d^	–	+	NA	010	–	48/32
E6	–	–	NA	New	347	–

*RT* PCR-ribotype, *ST* sequence type, MIC_0_: initial minimal inhibitory concentration to metronidazole (μg/ml), *MIC*_*S*_: minimal inhibitory concentration to metronidazole after repeated passages (μg/ml), *MZ* metronidazole, *NA* not applicable, *D* dog’s strain, *E* exotic species strain; ^a^isolate identification; ^b^non-toxigenic strains PCR; ^c^initial metronidazole-resistant isolates stability experiment results; ^d^metronidazole and multidrug-resistant isolates

The four (100%) toxigenic *C. difficile* positive dogs showed dysbiosis and faeces without mucus, while only 57 (67.8%) out of 84 of the *C. difficile* negative dogs showed this problem. However, this difference was not statistically significant (*p* = 0.3). No relationship was observed between the presence of *C. difficile* and any of the other factors considered (Table 1). None of the dogs presenting toxigenic *C. difficile* yielded a positive result for another enteric pathogen (data not shown).

**Fig. 1:**
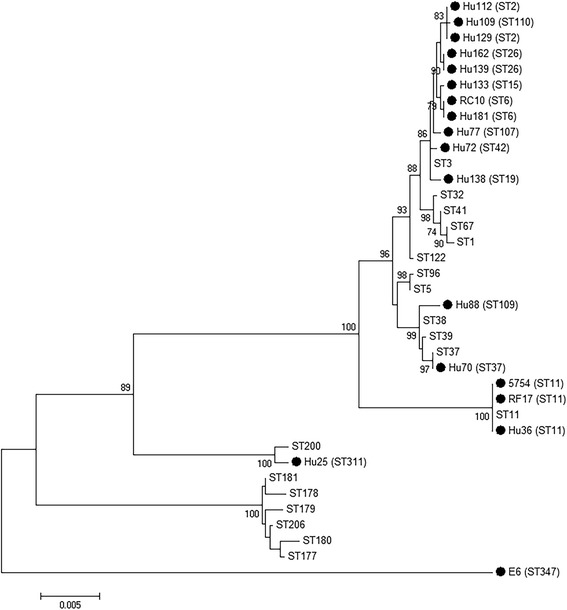
Molecular phylogenetic analysis (maximum likelihood method) from concatenated MLST alleles. C*lostridium difficile* isolates corresponding to our collection are showed with a circle. ST, sequence type; Hu, human isolate; RC, rat intestinal content isolate; 5754, sow vagina isolate; RF, environmental rat faeces isolate; E, exotic animal isolate

Twenty-four faecal samples derived from exotic species, 10 (41.7%) from diarrhoeic animals, 11 (45.8%) from non-diarrhoeic animals and three with unknown clinical data (12.5%). Fifteen (62.5%) were birds, mainly composed of different species of psittacines, 3 (12.5%) small mammals (lagomorphs, mustelids and rodents) and 6 (25%) sauropsid reptiles. Only one sample (4.2%; E6 strain, Table 2) belonging to a reptile (*Pogona vitticeps*) yielded a positive culture result for *C. difficile*. It was negative for all toxin genes tested (*tcdA*, *tcdB*, *cdtA* and *cdtB*) but also negative to the non-toxigenic PCR (see Additional file 1) and to the toxinotyping scheme (A1, A2, A3, B1, B2 and B3 fragments) (see Additional files 2 and 3). Interestingly, this isolate represented a new PCR-ribotyping type (Table 2) and belonged to ST347, which was recently proposed as a Clade C-III member [29] (Fig. 1).

### Antimicrobial susceptibility testing

All dog strains were susceptible to tetracycline and vancomycin (range between 0.064–0.38 μg/ml for vancomycin), whereas resistance to clindamycin, erythromycin, metronidazole and moxifloxacin varied (50%, 33.3%, 33.3% and 16.7% respectively) (Table 3). Two strains showed a MDR phenotype to clindamycin, erythromycin and metronidazole; resistance to metronidazole was unchanged and stable after a serial number of passages on antibiotic-free medium (Table 2). The MICs of the remaining strains (*n* = 4) to metronidazole varied between 0.19–0.38 μg/ml.

**Table 3:** In vitro activity of six antimicrobials against the *Clostridium difficile* dog isolates

Antimicrobial agent	Range (μg/ml)	Breakpoint^a^ (μg/ml)	Number resistant isolates (%)
Clindamycin	0′016–256	≥8^b^	3/6 (50)
Erythromycin	0′016–256	≥8	2/6 (33.3)
Metronidazole	0′016–256	≥32^b^	2/6 (33.3)
Moxifloxacin	0′02–32	≥8^b^	1/6 (16.7)
Tetracycline	0′016–256	≥8	0
Vancomycin	0′016–256	≥32	0

^a^The breakpoints for resistance established by the Clinical and Laboratory Standards Institute (CLSI) for anaerobic bacteria are those marked by ^b^ [50]. The breakpoint for tetracycline was ≥8 μg/ml [51]. The remaining breakpoints were based on the literature [7]

**Fig. 2:**
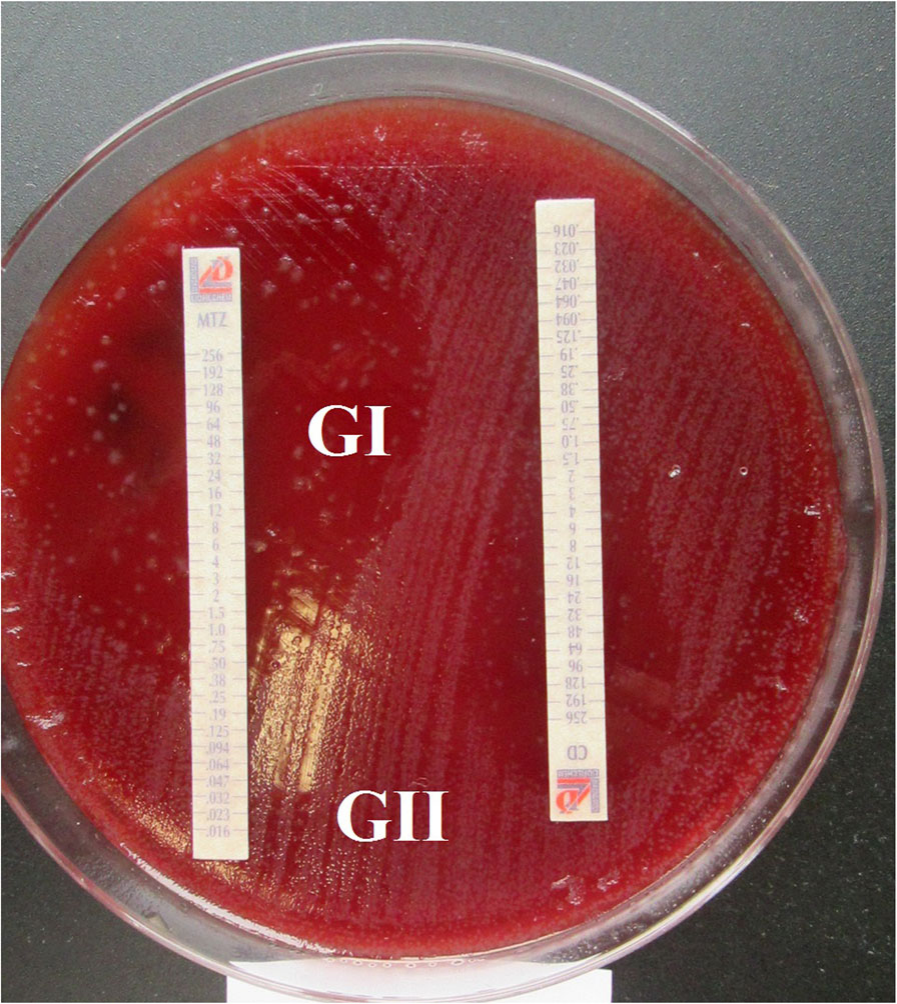
Metronidazole susceptibility test of Clostridium difficile D24 strain after 48 h of incubation. GI, growth I; GII, growth II

One strain (D24) was considered resistant (and stable) to metronidazole with a heterogeneous pattern. After the 24 h incubation time, the isolate showed a fully susceptible phenotype to metronidazole (MIC 0.38 μg/ml) (Fig. 2, growth II or GII; improved contrast image), but a subpopulation of tiny colonies (Fig. 2, growth I or GI) was present inside the initial inhibition halo exhibiting a higher MIC (8 μg/ml). MIC results remained unharmed after three extra days of incubation. After observing these results, the growth I and II were sub-cultured separately in blood agar without antibiotics in order to repeat the susceptibility test to metronidazole following the protocol described above. After 48 h of incubation, both isolates showed metronidazole resistance (MICs 128 and 192 μg/ml, GI and GII respectively). The metronidazole resistance stability test was performed only for GI (Table 2). A similar phenomenon was observed for this D24 isolate regarding its susceptibility to erythromycin and clindamycin. However, the subpopulations growing inside the initial halo reached a fully resistant phenotype to both antibiotics after 48 h of incubation. When the susceptibilities were repeated for the two different growths, both showed a fully resistant phenotype to these antimicrobials after 24 h.

The reptile *C. difficile* isolate (E6) was susceptible to all antimicrobial drugs (MICs range 0.19–2 μg/ml) tested except to tetracycline, for which a decreased susceptibility was found (MIC 6 μg/ml).

## Discussion

*Clostridium difficile* was isolated from 6.7% (6/90) of dogs with diarrhoea, but only four strains were toxigenic (66.7%). Overall, this prevalence was somewhat lower than that reported in previous studies of diarrheic dogs or dogs with various digestive disorders (29% -[30], 12.1% -[15] and 10% -[31]). However, these differences can be explained by variations of the study design (i.e. number and type of dogs included, geographical location, age distribution, etc.). They may be also due to the fact that samples were collected in veterinary clinics all around Spain and submitted to a veterinary diagnostic laboratory before arriving at our laboratory. It is well known that the survival rate of *C. difficile* may be compromised if faecal samples are stored under aerobic conditions [32]. It has also been reported that toxin-positive samples can yield a culture-negative result for *C. difficile* from dog faecal samples [9]. In addition, there were several limitations in this study to consider: the lack of the information on previous or ongoing antibiotic treatment data at the time of sampling.

Among the ribotypes detected in this study the most frequent was the epidemic RT014 (3/6) which has been widely isolated from CDI cases in humans in several European countries, including Spain [33]. This ribotype has been also isolated from different animal species and types of samples, including dogs, retail meat and water [34–36]. Only one out of the six isolates from this study belonged to the non-toxigenic ribotype 010 which has been largely associated with dogs [18, 37, 38] and occasionally isolated from humans [5]. These results add more evidence to a possible inter-species *C. difficile* transmission as previously observed other authors [39, 40]. However, to the author’s knowledge, there are no available data regarding the epidemiology of ribotypes 123 and 358 in Europe, which would suggest that they are not very common genotypes neither in humans nor in animals.

Metronidazole is the first choice to treat non-severe CDI in humans [41] and a very common treatment for CDI and *Giardia* spp. infections in dogs [16, 42]. Overall, the frequency of resistant *C. difficile* isolates to metronidazole is still low (0%–18%) [17], and even lower after laboratory strain manipulation [28]. In this study, a higher proportion of strains (33.3%, ribotypes 010 and 014) revealed stable resistance to metronidazole. An increase in the number of metronidazole-resistance *C. difficile* isolates has been observed for the last years in both humans and animals strains [17]. Interestingly, higher MIC averages to this drug are frequently associated with the non-toxigenic RT010 [19]. Thus, despite the low number of positive samples, these results could reflect this trend in dogs in Spain. In addition, these two isolates were also multi-drug resistant to clindamycin, erythromycin and metronidazole, a pattern already described in dogs [15, 18]. Overall, these results suggest that antimicrobial susceptibility surveillance programs should be implemented in *C. difficile* strains isolated from dogs since they could be a possible source of metronidazole-resistant and MDR *C. difficile*. However, in vitro results should be interpreted with caution due to the limited information available regarding breakpoints for anaerobic microorganisms of veterinary relevance, and the fact that the antimicrobial intestinal concentrations are not known for all cases.

The number of resistant isolates to moxifloxacin in this study was relatively low (1/6), which was expected due to the low use of this drug in companion animals in Spain and the low prevalence of resistance to this drug reported in previous studies in dogs and other animal species [43]. Although the use of clindamycin and erythromycin in companion animals in Spain is low [44], 50% and 33.3% of the isolates, respectively, were resistant to them. The resistance to clindamycin and erythromycin is common among *C. difficile* isolates in dogs [18]. This feature is mainly associated to *erm*B genes which are located in mobile elements, so these results probably reflect the spread of this determinant among *C. difficile* isolates regardless of their origin.

The results regarding strain D24 were unexpected. Heterogeneous and stable resistance to metronidazole was observed with high MIC values. The in vitro detection of subpopulations with reduced susceptibility to this antibiotic may be related to treatment failure observed in humans [28]. A similar phenomenon was observed on erythromycin and clindamycin susceptibility tests. The reason for these results is not clear and warrants further molecular studies.

The strain isolated from the lizard (E6) represents a new ribotype and it belongs to ST347 for which a new clade (C-III) was proposed [29]. A maximum likelihood tree was constructed using the E6 strain ST, sequence types from our *C. difficile* strain collection and from PubMLST database (Fig. 1), and it showed that E6 strain clade is completely apart from the rest of defined and proposed new clades described until the termination of this study [29, 45, 46]. E6 seems to be a non-toxigenic isolate (*tcdA*, *tcdB*, *cdtA* and *cdtB* negative results by PCR), but it yielded also a negative result in non-toxigenic PCR which allows thinking that it has not the traditional non-coding region which replaces the PaLoc as it has been described before [45, 47, 48]. Besides, the possible presence of toxin A and B genes remnants was analysed by toxinotyping with negative results as well. Therefore, it seems appropriate to perform whole genome sequencing analysis to verify the relationship of this isolate with *C. difficile* population and to examine its PaLoc insertion site organisation [48]. It is reasonable to assume that a new genotype could be introduced in Spain by an exotic animal species as has happened in the past with other *C. difficile* genotypes which have spread among different continents [49].

## Conclusions

In this study, 4.4% toxigenic *C. difficile* strains were isolated from diarrhoeic dogs. Since the ribotypes found in dogs are also commonly found in humans, it is possible that dogs may act as a source of contamination for human beings or vice versa. However, further studies are needed in particular to define the likely role of pets as vectors, especially in light of the spread of pet therapy practices. There is also a potential risk for the emergence of *C. difficile* strains resistant to the first line of antimicrobial drugs used for the treatment of CDI in people. Metronidazole is widely used in dogs, thus animals treated with this drug could be a possible source of MDR strains for humans. *Clostridium difficile* antimicrobial susceptibility surveillance programs should be advised in this animal species. Exotic animals can harbour uncommon *C. difficile* strains, different than those already established in the country of destination. The molecular results observed for E6 *C. difficile* strain, which can be also found in companion animal species, prompt the need for genome sequencing analyses to study this unusual isolate.

## Additional files

Additional file 1: *cdu1*-*cdd1* PCR (700 bp) agarose gel image from *Clostridium difficile* field isolates. Lane 1 *C. difficile* E6 strain, lanes 2–6 field isolates, lane 7 positive control, lane 8 negative control. M: 100 bp molecular mass (arrows point 500 bp fragment). (JPEG 1439 kb)

Additional file 2: *Clostridium difficile* field isolates and *Clostridium difficile* ATCC 43255 (positive control) toxinotyping in agarose gel. **a** A1 fragment, 3.1 kb: lane 1 E6 isolate, lanes 2–5 field isolates, lane 6 ATTC strain, lane 7 negative control. **b** A3 fragment, 3.1 kb: lane 1 ATCC strain, lane 2 E6 strain, lane 3 negative control. **c** B1 fragment, 3.1 kb: lanes 1 and 3 field isolates, lane 2 E6 strain, lane 4 ATCC strain, lane 5 negative control. M: 1 kb molecular mass (upper arrows point to 3 kb fragment and lower arrows point to 1 kb fragment) (JPEG 1765 kb)

Additional file 3: *Clostridium difficile* field isolates and *Clostridium difficile* ATCC 43255 (positive control) toxinotyping in agarose gel. Lanes 1–3 A2 fragment, 2 kb: ATTC strain, E6 strain and negative control respectively; lanes 4–6 B2 fragment, 2 kb: ATTC strain, E6 strain and negative control respectively; lanes 7–9 B3 fragment, 2 kb: ATTC strain, E6 strain and negative control respectively. M: 1 kb molecular mass (upper arrows point to 3 kb fragment and lower arrows point to 1 kb fragment) (JPEG 839 kb)

## Abbreviations

CD: *Clostridium difficile*
CDI: *Clostridium difficile* infection
D: Dog *C. difficile* isolate
E: Exotic animal *C. difficile* isolate
GI, GII: growth I and II respectively
Hu: Human *C. difficile* isolate
MDR: Multidrug resistance
MIC: Minimal inhibitory concentration
MIC_0_: Initial minimal inhibitory concentration to metronidazole
MIC_S_: Minimal inhibitory concentration to metronidazole after repeated passages
MLST: Multilocus sequence typing
MZ: Metronidazole
RC: Rat intestinal content *C. difficile* isolate
RF: Environmental rat faeces *C. difficile* isolate
RT: PCR-ribotype
ST: Sequence type

## References

[1] Rupnik M, Wilcox MH, Gerding DN (2009). *Clostridium difficile* infection: new developments in epidemiology and pathogenesis. Nat Rev Microbiol.

[2] Rodriguez-Palacios A, Borgmann S, Kline TR, LeJeune JT (2013). *Clostridium difficile* in foods and animals: history and measures to reduce exposure. Anim health res Rev.

[3] Taylor LH, Latham SM, Woolhouse M (2001). Risk factors for human disease emergence. Philos Trans R Soc Lond Ser B Biol Sci.

[4] Arroyo LG, Kruth SA, Willey BM, Staempfli HR, Low DE, Weese JS (2005). PCR ribotyping of *Clostridium difficile* isolates originating from human and animal sources. J Med Microbiol.

[5] Davies KA, Longshaw CM, Davis GL, Bouza E, Barbut F, Barna Z (2014). Underdiagnosis of *Clostridium difficile* across Europe: the European, multicentre, prospective, biannual, point-prevalence study of *Clostridium difficile* infection in hospitalised patients with diarrhoea (EUCLID). Lancet Infect Dis.

[6] Souza MJ (2011). One health: zoonoses in the exotic animal practice. Vet Clin North Am - Exot Anim Pract.

[7] Álvarez-Pérez S, Blanco JL, Martínez-Nevado E, Peláez T, Harmanus C, Kuijper E (2014). Shedding of *Clostridium difficile* PCR ribotype 078 by zoo animals, and report of an unstable metronidazole-resistant isolate from a zebra foal (*Equus quagga burchellii*). Vet Microbiol.

[8] Cave NJ, Marks SL, Kass PH, Melli AC, Brophy MA (2002). Evaluation of a routine diagnostic fecal panel for dogs with diarrhea. J Am Vet Med Assoc.

[9] Weese JS, Staempfli HR, Prescott JF, Kruth SA, Greenwood SJ, Weese HE (2001). The roles of *Clostridium difficile* and enterotoxigenic *Clostridium perfringens* in diarrhea in dogs. J Vet Intern Med.

[10] Marks SL, Rankin SC, Byrne BA, Weese JS (2011). Enteropathogenic bacteria in dogs and cats: diagnosis, epidemiology, treatment, and control. J Vet Intern Med.

[11] Schneeberg A, Rupnik M, Neubauer H, Seyboldt C (2012). Prevalence and distribution of *Clostridium difficile* PCR ribotypes in cats and dogs from animal shelters in Thuringia, Germany. Anaerobe.

[12] Berry AP, Levett PN (1986). Chronic diarrhoea in dogs associated with *Clostridium difficile* infection. Vet Rec.

[13] Marks SL, Kather EJ, Kass PH, Melli AC (2002). Genotypic and phenotypic characterization of *Clostridium perfringens* and *Clostridium difficile* in diarrheic and healthy dogs. J Vet Intern Med.

[14] Weese JS, Armstrong J (2003). Outbreak of *Clostridium difficile*-associated disease in a small animal veterinary teaching hospital. J Vet Intern Med.

[15] Orden C, Blanco JL, Álvarez-Pérez S, Garcia-Sancho M, Rodriguez-Franco F, Sainz A (2017). Isolation of *Clostridium difficile* from dogs with digestive disorders, including stable metronidazole-resistant strains. Anaerobe.

[16] Marks SL, Kather EJ (2003). Antimicrobial susceptibilities of canine *Clostridium difficile* and *Clostridium perfringens* isolates to commonly utilized antimicrobial drugs. Vet Microbiol.

[17] Spigaglia P (2016). Recent advances in the understanding of antibiotic resistance in *Clostridium difficile* infection. Ther Adv Infect Dis.

[18] Spigaglia P, Drigo I, Barbanti F, Mastrantonio P, Bano L, Bacchin C (2015). Antibiotic resistance patterns and PCR-ribotyping of *Clostridium difficile* strains isolated from swine and dogs in Italy. Anaerobe.

[19] Moura I, Spigaglia P, Barbanti F, Mastrantonio P (2013). Analysis of metronidazole susceptibility in different *Clostridium difficile* PCR ribotypes. J Antimicrob Chemother.

[20] Richardson C, Kim P, Lee C, Bersenas A, Weese JS (2015). Comparison of *Clostridium difficile* isolates from individuals with recurrent and single episode of infection. Anaerobe.

[21] Andrés-Lasheras S, Bolea R, Mainar-Jaime RC, Kuijper E, Sevilla E, Martín-Burriel I (2017). Presence of *Clostridium difficile* in pig faecal samples and wild animal species associated with pig farms. J Appl Microbiol.

[22] Rupnik M, Janezic S (2016). An update on *Clostridium difficile* toxinotyping. J Clin Microbiol.

[23] Griffiths D. *Clostridium difficile* MLST Home Page. https://pubmlst.org/cdifficile/. Accessed 13 October 2017.

[24] Griffiths D, Fawley W, Kachrimanidou M, Bowden R, Crook DW, Fung R (2010). Multilocus sequence typing of *Clostridium difficile*. J Clin Microbiol.

[25] Kimura M (1980). A simple method for estimating evolutionary rate of base substitutions through comparative studies of nucleotide sequences. J Mol Evol.

[26] Martín-Burriel I, Andrés-Lasheras S, Harders F, Mainar-Jaime RC, Ranera B, Zaragoza P (2017). Molecular analysis of three *Clostridium difficile* strain genomes isolated from pig farm-related samples. Anaerobe.

[27] Kumar S, Stecher G, Tamura K (2016). MEGA7: molecular evolutionary genetics analysis version 7.0 for bigger datasets. Mol Biol Evol.

[28] Peláez T, Cercenado E, Alcalá L, Marín M, Martín-López A, Martínez-Alarcón J (2008). Metronidazole resistance in *Clostridium difficile* is heterogeneous. J Clin Microbiol.

[29] Janezic S, Potocnik M, Zidaric V, Rupnik M (2016). Highly divergent *Clostridium difficile* strains isolated from the environment. PLoS One.

[30] Koene MGJ, Mevius D, Wagenaar JA, Harmanus C, Hensgens MPM, Meetsma AM (2012). *Clostridium difficile* in Dutch animals: their presence, characteristics and similarities with human isolates. Clin Microbiol Infect.

[31] Wetterwik K-J, Trowald-Wigh G, Fernström L-L, Krovacek K (2013). *Clostridium difficile* in faeces from healthy dogs and dogs with diarrhea. Acta Vet Scand.

[32] Weese JS, Staempfli HR, Prescott JF (2000). Survival of *Clostridium difficile* and its toxins in equine feces: implications for diagnostic test selection and interpretation. J Vet Diagnostic Investig.

[33] Freeman J, Vernon J, Morris K, Nicholson S, Todhunter S, Longshaw C (2015). Pan-European longitudinal surveillance of antibiotic resistance among prevalent *Clostridium difficile* ribotypes. Clin Microbiol Infect.

[34] Janezic S, Zidaric V, Pardon B, Indra A, Kokotovic B, Blanco JL (2014). International *Clostridium difficile* animal strain collection and large diversity of animal associated strains. BMC Microbiol.

[35] Rodriguez-Palacios A, Reid-Smith RJ, Staempfli HR, Daignault D, Janecko N, Avery BP (2009). Possible seasonality of *Clostridium difficile* in retail meat, Canada. Emerg Infect Dis.

[36] Zidaric V, Beigot S, Lapajne S, Rupnik Maja M (2010). The occurrence and high diversity of *Clostridium difficile* genotypes in rivers. Anaerobe.

[37] Álvarez-Pérez S, Blanco JL, Peláez T, Lanzarot MP, Harmanus C, Kuijper E (2015). Faecal shedding of antimicrobial-resistant *Clostridium difficile* strains by dogs. J Small Anim Pract.

[38] Keel K, Brazier JS, Post KW, Weese S, Songer JG (2007). Prevalence of PCR ribotypes among *Clostridium difficile* isolates from pigs, calves, and other species. J Clin Microbiol.

[39] Gould LH, Limbago B (2010). *Clostridium difficile* in food and domestic animals: a new foodborne pathogen. Clin Infect Dis.

[40] Stone NE, Sidak-Loftis LC, Sahl JW, Vazquez AJ, Wiggins KB, Gillece JD (2016). More than 50% of *Clostridium difficile* isolates from pet dogs in flagstaff, USA, carry toxigenic genotypes. PLoS One.

[41] Debast SB, Bauer MP, Kuijper EJ (2014). European Society of Clinical Microbiology and Infectious Diseases: update of the treatment guidance document for *Clostridium difficile* infection. Clin Microbiol Infect.

[42] Tangtrongsup S, Scorza V (2010). Update on the diagnosis and management of *Giardia* spp infections in dogs and cats. Top Companion Anim Med.

[43] Pirš T, Avberšek J, Zdovc I, Krt B, Andlovic A, Lejko-Zupanc T (2013). Antimicrobial susceptibility of animal and human isolates of *Clostridium difficile* by broth microdilution. J Med Microbiol.

[44] European Medicines Agency (2015). Sales of veterinary antimicrobial agents in 26 EU/EEA countries in 2013. European surveillance of veterinary antimicrobial consumption.

[45] Dingle KE, Elliott B, Robinson E, Griffiths D, Eyre DW, Stoesser N (2014). Evolutionary history of the *Clostridium difficile* pathogenicity locus. Genome Biol Evol.

[46] Knetsch CW, Terveer EM, Lauber C, Gorbalenya AE, Harmanus C, Kuijper EJ (2012). Comparative analysis of an expanded *Clostridium difficile* reference strain collection reveals genetic diversity and evolution through six lineages. Infect Genet Evol.

[47] Janezic S, Marín M, Martín A, Rupnik M (2015). A new type of toxin A-negative, toxin B-positive *Clostridium difficile* strain lacking a complete *tcdA* gene. J Clin Microbiol.

[48] Monot M, Eckert C, Lemire A, Hamiot A, Dubois T, Tessier C (2015). *Clostridium difficile*: new insights into the evolution of the pathogenicity locus. Sci Rep.

[49] He M, Miyajima F, Roberts P, Ellison L, Pickard DJ, Martin MJ (2013). Emergence and global spread of epidemic healthcare-associated *Clostridium difficile*. Nat Genet.

[50] CLSI. Performance standards for antimicrobial susceptibility testing; twenty-fourth informational supplement. CLSI document M100-S24. Wayne, PA: Clinical and Laboratory Standards Institute; 2014.

[51] Spigaglia P, Barbanti F, Mastrantonio P (2008). Tetracycline resistance gene *tet*(W) in the pathogenic bacterium *Clostridium difficile*. Antimicrob Agents Chemother.

