# Supplementary figures and images for: Preliminary studies on isolates of Clostridium difficile from dogs and exotic pets
Source: BMC Vet Res. 2018 Mar 9;14:77. doi: 10.1186/s12917-018-1402-7 (PMC5845233; doi:10.1186/s12917-018-1402-7)

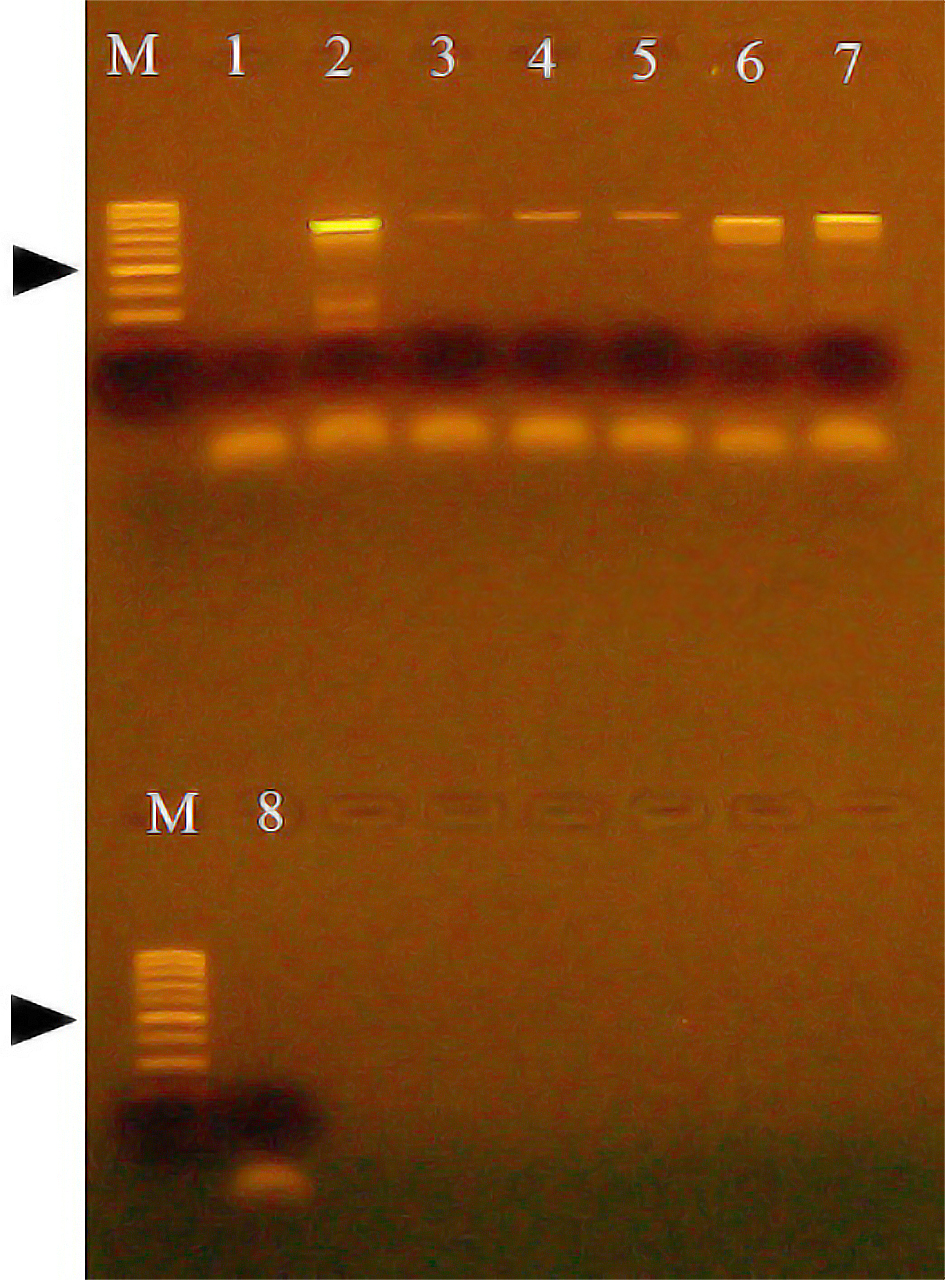

Supplement: Supplementary file 1 — cdu1-cdd1 PCR (700 bp) agarose gel image from Clostridium difficile field isolates. Lane 1 C. difficile E6 strain, lanes 2–6 field isolates, lane 7 positive control, lane 8 negative control. M: 100 bp molecular mass (arrows point 500 bp fragment). (JPEG 1439 kb) [file 12917_2018_1402_MOESM1_ESM.jpg]

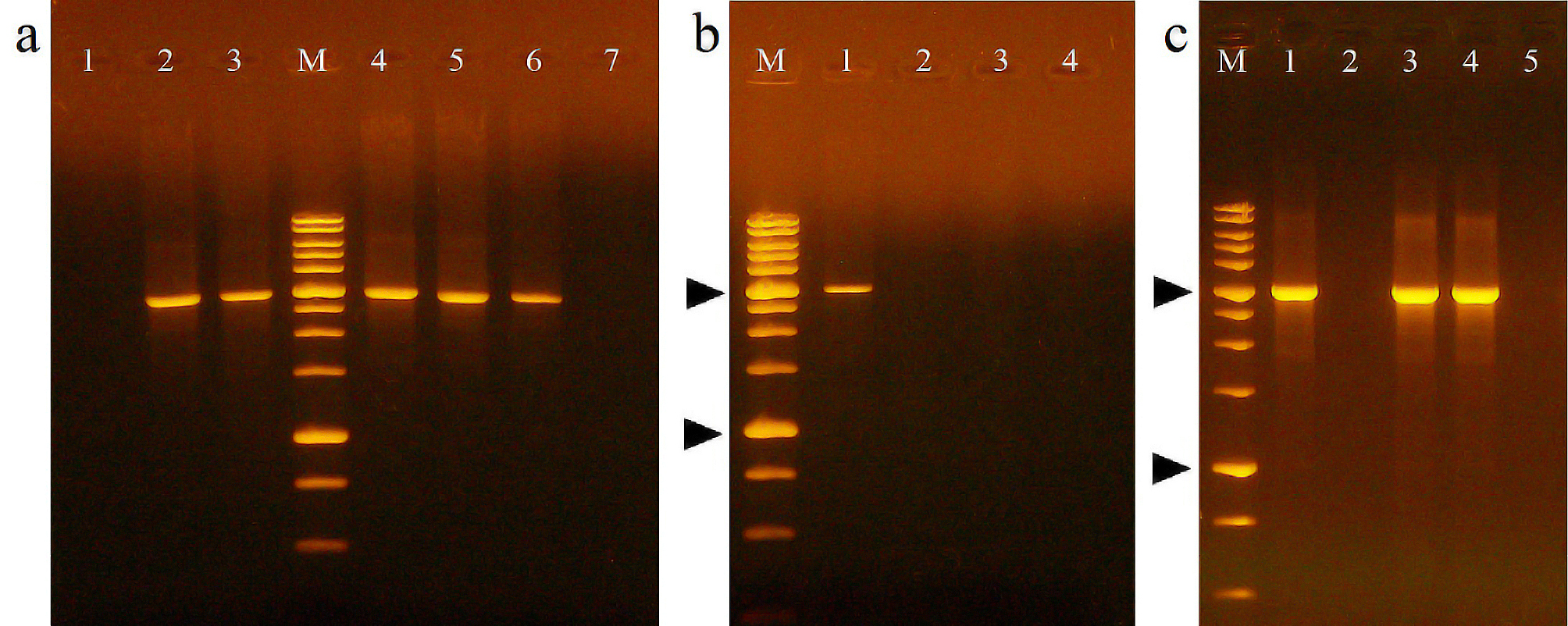

Supplement: Supplementary file 2 — Clostridium difficile field isolates and Clostridium difficile ATCC 43255 (positive control) toxinotyping in agarose gel. a A1 fragment, 3.1 kb: lane 1 E6 isolate, lanes 2–5 field isolates, lane 6 ATTC strain, lane 7 negative control. b A3 fragment, 3.1 kb: lane 1 ATCC strain, lane 2 E6 strain, lane 3 negative control. c B1 fragment, 3.1 kb: lanes 1 and 3 field isolates, lane 2 E6 strain, lane 4 ATCC strain, lane 5 negative control. M: 1 kb molecular mass (upper arrows point to 3 kb fragment and lower arrows point to 1 kb fragment) (JPEG 1765 kb) [file 12917_2018_1402_MOESM2_ESM.jpg]

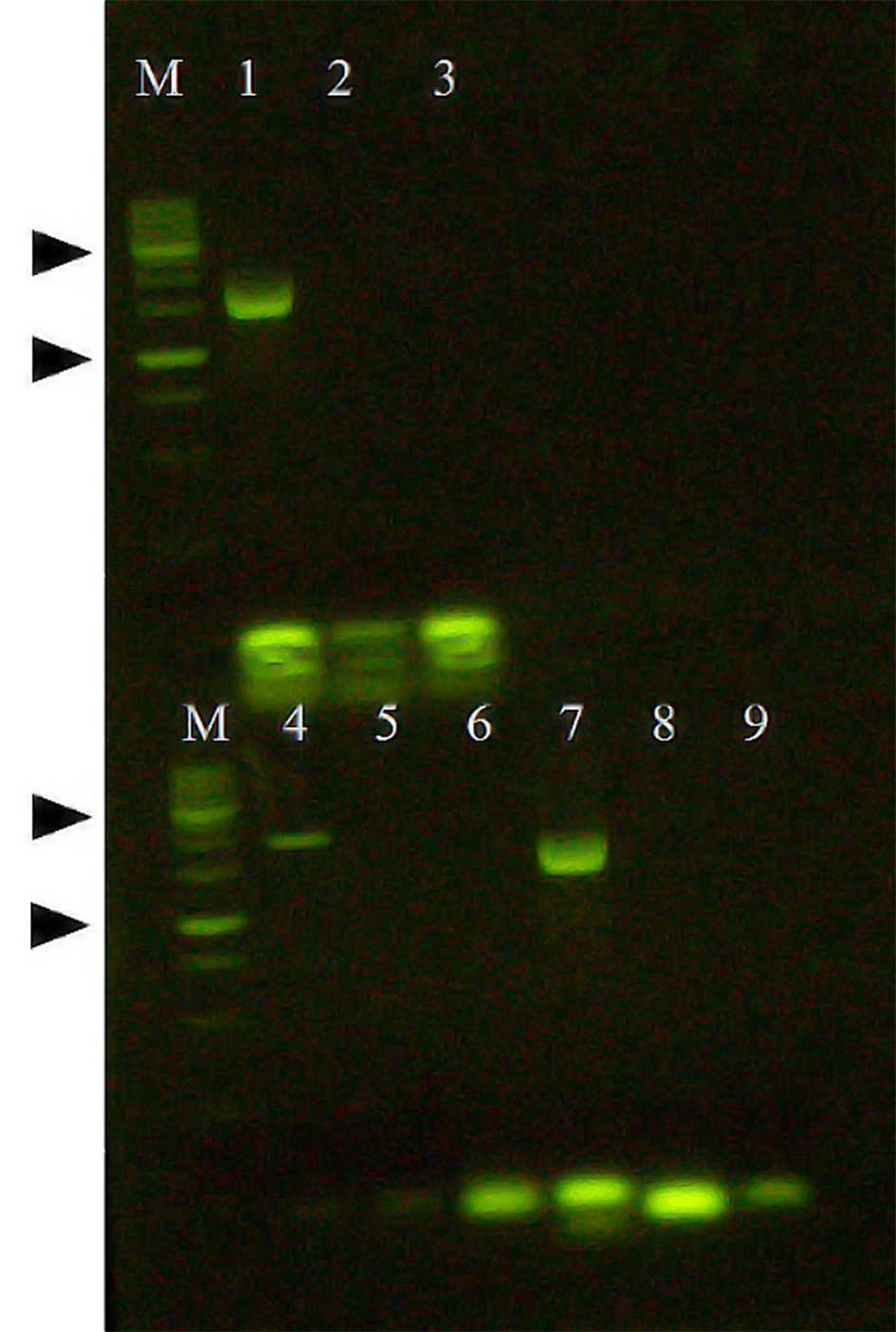

Supplement: Supplementary file 3 — Clostridium difficile field isolates and Clostridium difficile ATCC 43255 (positive control) toxinotyping in agarose gel. Lanes 1–3 A2 fragment, 2 kb: ATTC strain, E6 strain and negative control respectively; lanes 4–6 B2 fragment, 2 kb: ATTC strain, E6 strain and negative control respectively; lanes 7–9 B3 fragment, 2 kb: ATTC strain, E6 strain and negative control respectively. M: 1 kb molecular mass (upper arrows point to 3 kb fragment and lower arrows point to 1 kb fragment) (JPEG 839 kb) [file 12917_2018_1402_MOESM3_ESM.jpg]
